# Measuring variability in trophic status in the Lake Waco/Bosque River Watershed

**DOI:** 10.1186/1754-1611-2-1

**Published:** 2008-01-11

**Authors:** Angela D Rodriguez, Marty D Matlock

**Affiliations:** 1 Bury+Partners, Engineering Solutions, 221 West Sixth Street, Suite 600, Austin, Texas, USA; 2 Department of Biological and Agricultural Engineering, University of Arkansas, Fayetteville, AR, USA

## Abstract

**Background:**

Nutrient management in rivers and streams is difficult due to the spatial and temporal variability of algal growth responses. The objectives of this project were to determine the spatial and seasonal *in situ *variability of trophic status in the Lake Waco/Bosque River watershed, determine the variability in the lotic ecosystem trophic status index (LETSI) at each site as indicators of the system's nutrient sensitivity, and determine if passive diffusion periphytometers could provide threshold algal responses to nutrient enrichment.

**Methods:**

We used the passive diffusion periphytometer to measure *in-situ *nutrient limitation and trophic status at eight sites in five streams in the Lake Waco/Bosque River Watershed in north-central Texas from July 1997 through October 1998. The chlorophyll *a *production in the periphytometers was used as an indicator of baseline chlorophyll *a *productivity and of maximum primary productivity (MPP) in response to nutrient enrichment (nitrogen and phosphorus). We evaluated the lotic ecosystem trophic status index (LETSI) using the ratio of baseline primary productivity to MPP, and evaluated the trophic class of each site.

**Results:**

The rivers and streams in the Lake Waco/Bosque River Watershed exhibited varying degrees of nutrient enrichment over the 18-month sampling period. The North Bosque River at the headwaters (NB-02) located below the Stephenville, Texas wastewater treatment outfall consistently exhibited the highest degree of water quality impact due to nutrient enrichment. Sites at the outlet of the watershed (NB-04 and NB-05) were the next most enriched sites. Trophic class varied for enriched sites over seasons.

**Conclusion:**

Seasonality played a significant role in the trophic class and sensitivity of each site to nutrients. Managing rivers and streams for nutrients will require methods for measuring *in situ *responses and sensitivities to nutrient enrichment. Nutrient enrichment periphytometers show significant potential for use in nutrient gradient studies.

## Background

Algae have been used to determine stream ecosystem impacts from human activities for more than 50 years [1]. Increased loading of nutrients into streams and lakes has become one the major environmental problems facing society today [2,3]. Nutrients are the leading cause for the degradation of water quality in lakes and estuaries in the US and second to siltation among the Nation's rivers and streams [3]. Nutrients released from runoff into an aquatic ecosystem stimulate algal growth and hence accelerates the eutrophication of surface water [4,2,5]. Nutrient enrichment in streams and rivers is a consequence of urban, industrial, and agricultural use of fertilizers, particularly nitrogen (N) and phosphorus (P), and their subsequent disposal [3].

Algal growth, like plants, is limited by the nutrient in least supply relative to their needs [6,7]. If algal growth can be shown to increase in response to nutrient enrichment, then that nutrient is the nutrient that limits algal growth [8]. Algal species in freshwater systems tend to be limited by inorganic phosphorous because its proportional abundance is lower in the lithosphere (upper geologic strata) than in plant tissue [8,9]. Algae in marine systems are presumed to be nitrogen limited, and estuarine systems are presumed to be enriched by both nitrogen and phosphorus [10].

However, these overly simplistic generalizations do not provide meaningful guidance in managing water quality in a specific water body. When a water body is enriched with nutrients, the kinetics of nutrient enrichment can shift over time and space rather quickly [11]. Lotic (flowing water) ecosystems are characterized as disturbance-dominated systems. In lotic ecosystems many variables can contribute to algal biomass growth and accumulation, including flow/scour intensity, canopy cover/light, temperature, substrate size/composition, sediment content in the water, rate of grazing pressure from macroinvertebrates and fish, and other variables [11].

Allen and Hershey [12] determined that nutrient limitation of algal biomass was seasonally dynamic and thus nutrient flux within the watershed was influenced by these numerous processes. The periphytic community is the principle benthic community to accumulate and retain dissolved nutrients and toxicants [13-15,5], and thus is a reasonable bio-indicator of stream nutrient status. However, detecting algal nutrient responses independent of these many other variables is difficult, because of the variables previously mentioned.

Management of water quality for nutrients and algal growth at the watershed (catchment) level has been a difficult challenge since the Clean Water Act was implemented over 30 years ago. The United States Environmental Protection Agency has developed nutrient criteria recommendations for managing water quality in rivers and streams [16]. The USEPA suggested trophic classification for benthic chlorophyll production in Ecoregion V based upon mean and maximum benthic (periphytic) algae chlorophyll *a *production [16]. Measuring these benthic chlorophyll levels requires a method determining periphytic responses to nutrients in streams over time and space, independent of these confounding variables.

Water quality throughout the Lake Waco/Bosque River watershed has been in decline due to nutrient enrichment [17]. In the North Bosque River sub-watershed, dairy waste was identified as a major source of nutrients contributing to water quality problems [18]. High in-stream phosphorus concentrations were associated with drainages dominated by dairy waste application fields in the upper portion of the North Bosque River [19]. In the Middle and South Bosque Rivers, elevated nitrogen levels associated with intensive row-crop agriculture were also considered a threat to maintaining water quality in these two rivers [20]. These nutrients degraded water quality in Lake Waco at the outlet of the Bosque River Watershed, the primary supply of drinking water for the City of Waco, Texas (with a population of about 140,000 people). Matlock *et al.*[15] determined that the algal growth-limiting nutrients in the system varied down the stream gradient during a season; the extent and variability of this variation remained uncharacterized.

The lotic ecosystem trophic status index (LETSI) was developed as a tool for making comparisons of stream biotic response to nutrients [15]. The underlying assumption of this index is that algal growth in the presences of excess nitrogen and phosphorus will result in some maximum potential productivity (MPP) of chlorophyll *a *at a given site over a given sampling period. The MPP represents the level of periphytic growth (measured as chlorophyll *a *production) that should occur when nutrients are not limiting. Of course, primary productivity (carbon fixed per unit time per area or mass of biomass) is NOT the same as chlorophyll *a *production, but chlorophyll *a *is the parameter most often utilized in management of water resources. The LETSI is then defined as the ratio of the baseline algal chlorophyll *a *production rate to the MPP. Theoretically, the LETSI ranges from 0 to 1.0, representing the proportion of the control chlorophyll production rate to the chlorophyll production rate that could occur over the sample period (14 days) under in situ conditions (flow, temperature, light, turbidity, etc.). Thus, LETSI represents a potential metric for measuring stream nutrient status in the context of all the other variables that affect algal growth at a given stream site. The trophic classification recommended by USEPA [16] for Eco-Region 5 was applied to these systems, where Control treatment chlorophyll a of 2.0 μg/cm^2 ^(mean) and 6 μg/cm^2 ^(maximum) represented the transition from oligotrophic to mesotrophic, and 7.0 μg/cm^2 ^(mean) and 20 μg/cm^2 ^(maximum) from mesotrophic to eutrophic classification.

The objectives of this project were to determine the spatial and seasonal *in situ *periphytic trophic status in the Lake Waco/Bosque River watershed, determine the seasonal variability in the lotic ecosystem trophic status index (LETSI) at each site as indicators of the Bosque River' trophic classification [15,16], and determine if algae responded to threshold nutrient enrichment *in situ *using the passive diffusion periphytometer. The hypotheses tested were:

**H**_**o1**_: Stream trophic status did not vary throughout the seasons across sites; and

**H**_**o2**_: Periphytic chlorophyll *a *growth in response to nutrient enrichment from the passive diffusion periphytometer was independent of nutrient enrichment concentration.

## Methods

### Site descriptions

**Figure 1:**
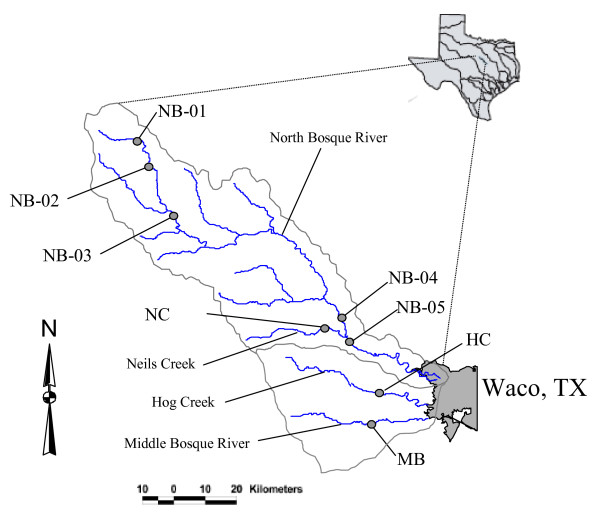
Stream sampling locations in the Bosque Watershed, North Central Texas.

The Bosque River Watershed covers about 430,000 ha in central Texas, 74% of which is represented by the drainage of the North Bosque River (Fig. 1). Other major drainages in the Bosque River Watershed include Hog Creek and Middle and South Bosque Rivers. The northern two-thirds of the Bosque River Watershed is in the Central Plains Ecoregion, while the southern third is in the Blackland Prairie Ecoregion of Texas [17].

Eight stream sample sites on five streams in the Lake Waco/Bosque River Watershed, each with different nutrient characteristics, were targeted to determine the limiting nutrient and to compare lotic ecosystem trophic status under varying conditions during July, 1997 and April, May, August and October 1998. The stream sample locations (Fig. 1) were selected throughout the Lake Waco/Bosque River watershed to correspond with water-quality sampling sites established by the Texas Institute for Applied Environmental Research (TIAER) [18]. Progressing down-watershed, the first site (NB-01) is on the North Bosque River above Stephenville, Texas (pop. 14,000). This is a third order stream with silt-clay substrate, steep banks, low base flow, and closed canopy. The stream is 3–4 m wide and 1.5 m deep at Site NB-01. About 12 percent of the land area above Site NB-01 is used for dairy waste application. The next site (NB-02) is on the North Bosque River below the Stephenville Wastewater Treatment Plant (WWTP), about eight river km below Site NB-01. The North Bosque River is a fourth order stream at this location, 5–6 m wide and 1.5 m deep, silt-clay bottomed with open canopy. While dairy production is an important feature in the drainage area above NB-02, the predominant source of nutrients at base flow at NB-02 is the Stephenville WWTP [19]. NB-03 is located on the North Bosque River at Hico, Texas (pop. 1,400). The North Bosque River is still a fourth order stream at NB-04 near Clifton, Texas (pop. 3,400), about 80 river km below NB-03 and 64 km above Lake Waco. The lowest site in the North Bosque River watershed was NB-05 at Valley Mills, Texas, about 12 km below NB-04 and 52 km above Lake Waco. The drainage area for NB-05 is approximately 254,000 ha, with about 4 percent of land area designated for dairy waste application. There are four small municipal WWTPs contributing nutrients to the river above NB-05: the cities of Stephenville, Hico, Irredell, and Meridian.

The Middle Bosque subwatershed covers approximately 31,000 ha, and the land use is predominantly cropland and rangeland. The sample site (MB) has episodic nutrient loading associated with runoff [19]. This sample site is located about 19 km upstream from Lake Waco and east of the city of Crawford (pop. 600). The river is third-order, about 1.5 m deep and 10 m wide, with gravel, cobble, and boulder substrate. The canopy is open, and there is very little sediment in the water. Hog Creek (HC) is a tributary to the Middle Bosque River. The sample site was selected so geomorphologic characteristics were similar to the Middle Bosque River site. The final site is on Neil's Creek (NC); this is the least nutrient enriched stream in the watershed and is considered the reference water body for this study. Neil's Creek is very similar in geomorphology to MB; the primary land uses in the drainage above NC are forest and native rangeland.

### Weather conditions and seasonal sampling strategy

Flow in the Bosque River stream systems during the sample period (1997–1998) was characterized by high intensity rainfall events in February 1997 and March 1998 [19], representing the highest and third-highest monthly flows on record, respectively, for the Bosque River. Thus, nutrients in the streams during this period were from both point and nonpoint sources [18], and represented a period of high disturbance.

We measured the periphytic nutrient trophic status (limiting nutrient) in streams and rivers at eight locations in the watershed over seasons using in situ passive diffusion periphytometers [15]. The period of measure was characterized by extreme hydrologic events, including a severe drought (50 year return period) and a severe flood (25 year event) [19]. Low flow conditions and scouring flows drive periphytic community structures in this system. We selected sample periods to measure periphytic biomass response to nutrients during July 1997 [15], and April, May, July, and October 1998, to capture nutrient-biotic dynamics during spring, summer, and fall conditions. Many deployments were lost due to inadequate flow or flood conditions during this period. For the analysis, July-August was considered Late Summer, October was considered Fall, and April-May was considered Spring. Due to significant deployment losses, site comparisons required grouping deployments to make near-complete sets.

### Passive diffusion periphytometers

**Figure 2:**
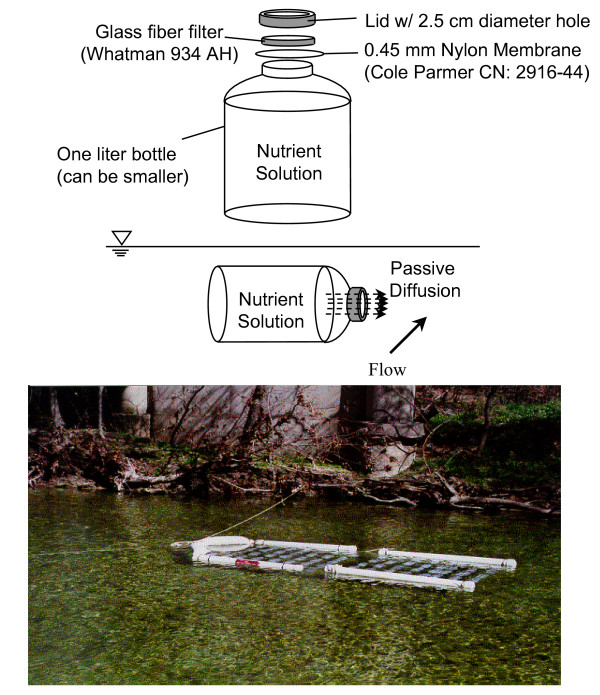
Passive diffusion (Matlock) periphytometer components.

Limiting nutrients (nitrogen, N and/or phosphorus, P) were determined for each stream site using passive diffusion periphytometers constructed of a 0.45 micron nylon membrane filter (Cole Parmer CN 2916-44) as a biofilter and Whatman 934-AH glass fiber filter as the growth substrate, attached to the top of a 1-liter low density polyethylene container with a 2.5 inch diameter hole cut in the lid (Fig. 2) [15]. The bottles were filled with the treatment nutrient solution, and attached to a floating rack. The four nutrient enrichment treatments were:

1. Control (C), consisting of deionized water, with a nominal conductivity of 30 μS/cm;

2. Nitrate (N), consisting of a solution of 0.35 mM (30 ppm) NaNO_3 _in de-ionized water;

3. Phosphate (P), consisting of a solution of 0.11 mM (30 ppm) of Na_2_HPO_4_-7H_2_O in deionized water;

4. Nitrate and Phosphate (N+P), consisting of a solution of 30 ppm NaNO_3 _and 30 ppm of Na_2_HPO_4_-7H_2_O in deionized water.

### Phosphorus gradient measurements

We deployed a gradient of phosphorus treatments in passive diffusion periphytometers to determine the critical concentration, or that concentration which elicits a significant algal biomass production response, at NB-03 and NB-05 during the summer condition (July, 1998). The concentrations were designed to simulate the following in-stream phosphorus concentrations, and were calculated as the instantaneous average mass flux through the membrane volume (1.56 cm^3^). The estimated concentrations, in μg/l PO4-P, were: P1 – 20, P2 – 45, P3 – 90, P4 – 200, and P5 – 500.

### Experimental design

The passive diffusion periphytometer treatments were arranged in a randomized block design consisting of a treatment array of four treatments per block, and ten replicates of each block per site. Each treatment array of forty periphytometers was supported on an iron wire frame, attached to PVC pontoons, and anchored in the middle of the river at the sample site. The periphytometers were attached to the wire frame with growth surfaces perpendicular to the water surface and parallel to stream flow [15]. The algal growth surfaces were protected from fish and macro-invertebrate grazing by placing an aluminum screen (8 mesh, or approximately 3 wires per cm, 0.7 mm diameter wire) over top of each periphytometer, approximately 5 cm from the glass fiber filter growth surfaces.

At the end of the 14-day growth period, the colonized glass fiber filters were placed in 5 ml of 90 percent acetone solution saturated with magnesium carbonate at 5°C, wrapped in aluminum foil, and transported to the laboratory for analysis. Chlorophyll was extracted from the filters for direct measurement in the laboratory using EPA Standard Method 10200H.3 [21]. Chlorophyll *a *from each filter sample was expressed as mass (μg) per unit of exposed surface area of the filter (6.6 cm^2^) for comparison. Mean chlorophyll *a *concentrations for all treatments across sites were compared using Student-Newman-Keuls' (SNK) and Waller – Duncan K Ratio (WD) tests (α = 0.05) in SAS/STAT^® ^[22]. The SNK tests are robust in detecting differences in means among many treatments using a step-down multiple comparisons procedure with critical values based on the Studentized range distribution, yet are conservative with regards to Type 2 errors [23,24]. The SNK procedure controls False Discovery Rate (FDR) Type 1 Errors typically associated with multiple comparisons [24]. The WD is used often in plot studies with high variability and low replicate numbers (n) [20]. Unequal replicates due to sample loss were corrected using the second approximation method as described by Steel and Torrie [25].

## Results and Discussion

### Seasonal differences in nutrient responses and LETSI

During the July 1997 sampling event, the periphytic biomass production in Neil's Creek (NC), the reference sub-watershed, was phosphorus limited, MB and NB-04 were co-limited by nitrogen (N) and phosphorus (P); the periphytic community at NB-01 and NB-02 was limited by something other than nutrients (perhaps light) (Table 1). According to the SNK grouping (α = 0.05) base chlorophyll *a *productivity at NC of 0.48 μg cm^-2^was not significantly different from sites NB-01 (0.88 μg cm^-2^), and MB (0.47 μg cm^-2^). Similarly, the MPP at site MB (5.87 μg cm^-2^) was not significantly different from sites NB-02 (5.19 μg cm^-2^), and NB-04 (5.73 μg cm^-2^). Site NB-02, the North Bosque River below the Stephenville WWTP, was one of the most nutrient-enriched sites of the five sites measured (Table 1).

**Table 1:** Student-Newman-Keuls' (SNK) Test Comparison of Chlorophyll *a *concentrations for Control, Nitrogen (N), Phosphorus (P), and Nitrogen plus Phosphorus (N + P) treatments using Passive Diffusion Periphytometer in the Bosque River Watershed during the period of July 17 – 30, 1997 (from Matlock et al., 1999, included for comparison).

Site	Treatment	Number of Replicates	Mean Chl. *a *(μg cm^-2^)	Standard Deviation (μg cm^-2^)	SNK Group (α = 0.05)				
**NB-01**	Control	10	0.88	0.60				D	E
	N	10	0.95	0.38				D	E
	P	9	1.06	0.53				D	E
	N + P	10	0.98	0.53				D	E
**NB-02**	Control	10	4.58	0.86	A				
	N	10	5.10	1.61	A	B			
	P	9	6.22	1.91	A	B			
	N + P	10	5.19	1.86	A	B			
**NB-04**	Control	7	1.73	0.37			C	D	
	N	5	2.08	0.38			C	D	
	P	9	2.49	0.51			C		
	N + P	8	5.73	1.36	A	B			
**MB**	Control	10	0.47	0.20					E
	N	9	0.52	0.08					E
	P	9	0.90	0.32				D	E
	N + P	10	5.87	1.93	A	B			
**NC**	Control	10	0.48	0.14					E
	N	10	0.59	0.17					E
	P	9	2.70	1.16			C		
	N + P	10	2.67	0.82			C		

The Lotic Ecosystem Trophic Status Indices measured at these five sites ranged from 0.08 to 0.90 (Table 2). Of the sites measured during this sample period, only NB-02 was mesotrophic; all others were oligotrophic. Sites NB-01 and NB-02 were at MPP indicating that additional nutrient loads would not shift their trophic status. With the exception of NC, the reference stream, all sites had the same MPP, and thus the potential to be mesotrophic if nutrients were available. Note that while sites NB-01 and NB-02 were both at MPP, NB-02 base productivity was over 5 times greater than NB-01. Site NB-02 was immediately below the outfall of the Stephenville WWTP, and was characterized by wide channel and diminished canopy cover compared with Site NB-01.

**Table 2:** Comparison of Lotic Ecosystem Trophic Status Indices (LETSIs) and Trophic Classifications using Passive Diffusion Periphytometers in the Bosque River Watershed for all sites sampled during the indicated periods.

Date	Site	Number of Replicates	Control Chl. *a *(ug cm^-2^)*	N+P Chl. *a *(ug cm^-2^)	LETSI	Ecoregion 5 Trophic Classification^#^
July 17 – 30, 1997	**NB-01**	10	0.88	0.98	0.90	**O**
	**NB-02**	10	4.58	5.19	0.88	**M**
	**NB-04**	10	1.73	5.73	0.30	**O**
	**NC**	10	0.48	2.67	0.08	**O**
	**MB**	10	0.47	5.87	0.18	**O**
April 5 – 19, 1998	**NC**	10	0.71	1.21	0.59	**O**
	**MB**	10	0.56	0.91	0.61	**O**
May 11 – 26, 1998	**NB-01**	8	0.40	0.37	>1.0	**O**
	**NB-02**	9	9.02	8.07	>1.0	**E**
	**NB-04**	9	2.50	4.41	0.57	**M**
	**NB-05**	9	2.69	6.13	0.44	**M**
	**HC**	7	1.46	1.16	>1.0	**O**
	**MB**	8	0.83	6.35	0.13	**O**
July 30 – August 13, 1998	**NB-04**	8	0.49	4.55	0.11	**O**
	**NB-05**	9	0.99	2.15	0.46	**O**
October 10 – 24, 1998	**NB-01**	7	0.80	0.73	1.10	**O**
	**NB-02**	6	1.94	1.50	1.30	**O**
	**NB-03**	7	0.61	1.56	0.39	**O**

*Measured as chlorophyll *a *production (μg cm^-2^) over 14 days on a control surface.

^# ^Trophic Classification defined by USEPA Eco-Region 5 (14), O = Oligotrophic, M = Mesotrophic, E = Eutrophic

During the April 1998 sampling period, site NC was phosphorous limited and site MB-060 was phosphorus enriched, exhibiting no nutrient limitation (Table 3). The base primary production at NC (0.71μg cm^-2^) was not significantly different than MB (0.56 μg cm^-2^) (Table 3, SNK Group C). The LETSI values indicate that both sites NC and MB were at approximately 60 percent of maximum primary productivity, yet site MB did not yield increased periphytic biomass with increased N or P.

**Table 3:** Student-Newman-Keuls' (SNK) Test Comparison of Chlorophyll *a *concentrations for Control, Nitrogen (N), Phosphorus (P), and Nitrogen plus Phosphorus (N + P) treatments using Passive Diffusion Periphytometer in the Bosque River Watershed during the period of April 5–19, 1998.

Site	Treatment	Number of Replicates	Mean Chl. *a *(μg cm^-2^)	Standard Deviation (μg cm^-2^)	SNK Group (α = 0.05)		
**NC**	Control	10	0.71	0.26		B	C
	N	10	0.89	0.55		B	C
	N+P	10	1.21	0.60	A	B	
	P	10	1.36	0.54	A		
**MB**	Control	10	0.56	0.27			C
	N	10	0.73	0.24		B	C
	N+P	10	0.91	0.63		B	C
	P	10	0.85	0.40		B	C

Six sites were sampled during May 1998 (Table 4). The Bosque River below the WWTP (NB-02) demonstrated the greatest MPP (9.02 μg cm^-2^). The baseline productivity was significantly different than all of the other sites monitored during this period. This site was not nutrient limited. The North Bosque River north of the WWTP (NB-01) and Hog Creek (HC) were at maximum potential productivity, while their production rates were five to 15 times less than NB-02. Sites NB-04 and NB-05 were phosphorous limited (Table 4), reflecting increased assimilative capacity for P. The Middle Bosque River was co-limited with a LETSI value of 13 percent (Table 2). Site NB-01 was oligotrophic, but at NB-02 (after the WWTP) the Bosque River became eutrophic. It was still mesotrophic at NB-04 and NB-05, over 100 km downstream of NB-02. The nonpoint source streams (HC and MB) were oligotrophic during that period (Table 2). Sites NB-03 and NB-05 were co-limited by phosphorus in late summer (July 1998, Table 5).

**Table 4:** Student-Newman-Keuls' (SNK) Test Comparison of Chlorophyll *a *concentrations for Control, Nitrogen (N), Phosphorus (P), and Nitrogen plus Phosphorus (N + P) treatments using Passive Diffusion Periphytometer in the Bosque River Watershed during the period of May 11–26, 1998.

Site	Treatment	Number of Replicates	Mean Chl. *a *(μg cm^-2^)	Standard Deviation (μg cm^-2^)	SNK Group (α = 0.05)					
**NB-01**	Control	8	0.40	0.16						F
	N	9	0.36	0.28						F
	N+P	9	0.37	0.13						F
	P	9	0.28	0.06						F
**NB-02**	Control	9	9.02	6.95		B	C			
	N	9	10.09	6.76	A	B				
	N+P	8	8.07	6.69		B	C	D		
	P	8	12.64	5.11	A					
**NB-04**	Control	9	2.50	0.70					E	F
	N	8	3.31	1.38					E	F
	N+P	7	4.41	1.77				D	E	F
	P	8	4.88	1.32				D	E	F
**NB-05**	Control	9	2.69	0.80					E	F
	N	8	3.43	2.44					E	F
	N+P	8	6.13	2.54			C	D	E	
	P	8	6.07	2.59			C	D	E	
**HC**	Control	7	1.46	0.48						F
	N	8	1.19	0.51						F
	N+P	8	1.16	0.58						F
	P	9	1.07	0.64						F
**MB**	Control	8	0.83	0.13						F
	N	9	1.33	0.82						F
	N+P	8	6.35	2.94			C	D	E	
	P	9	2.28	2.22					E	F

**Table 5:** Student-Newman-Keuls' (SNK) Test Comparison of Chlorophyll *a *concentrations for Control, Nitrogen (N), Phosphorus (P), and Nitrogen plus Phosphorus (N + P), and phosphorus gradient treatments (P1–P5, representing progressively higher P concentrations) using Passive Diffusion Periphytometer in the Bosque River Watershed during the period of July 30 – August 13, 1998.

Site	Treatment	Number of Replicates	Mean Chl. *a *(μg cm^-2^)	Standard Deviation (μg cm^-2^)	SNK Group (α = 0.05)		
**NB-03**	C	8	0.49	0.24			C
	N	9	0.86	0.35			C
	N+P	9	4.55	1.92	A		
	P1	7	0.67	0.17			C
	P2	7	0.71	0.24			C
	P3	9	0.67	0.24			C
	P4	9	0.85	0.42			C
	P5	9	0.79	0.19			C
**NB-05**	C	9	0.99	0.26			C
	N	9	1.02	0.42			C
	N+P	8	2.15	0.50		B	
	P1	9	1.08	0.34			C
	P2	9	1.13	0.37			C
	P3	9	1.17	0.36			C
	P4	8	0.89	0.34			C
	P5	9	1.99	0.59		B	

The final sampling event, October 10–24, 1998, was restricted to Sites NB-01, NB-02, and NB-03. Site NB-01 was not nutrient limited (Table 6). The baseline productivity at site NB-02 was significantly higher than NB-01, though this site was not nutrient limited either. Site NB-03 was co-limited as before (July, Table 5), suggesting phosphorus enrichment. The LETSI value for NB-03 was 39 percent, while the LETSI values at the other two sites exceeded one (Table 2). All sites were oligotrophic during that period.

**Table 6:** Student-Newman-Keuls' (SNK) Test Comparison of Chlorophyll *a *concentrations for Control, Nitrogen (N), Phosphorus (P), and Nitrogen plus Phosphorus (N + P) treatments using Passive Diffusion Periphytometer in the Bosque River Watershed during the period of October 10–24, 1998.

Site	Treatment	Number of Replicates	Mean Chl. *a *(μg cm^-2^)	Standard Deviation (μg cm^-2^)	SNK Group (α = 0.05)	
**NB-01**	Control	7	0.80	0.34		B
	N	7	0.78	0.30		B
	N+P	6	0.73	0.20		B
	P	7	0.73	0.23		B
**NB-02**	Control	6	1.94	1.19	A	B
	N	7	1.50	0.75	A	B
	N+P	6	1.50	1.09	A	B
	P	7	1.54	0.85	A	B
**NB-03**	Control	7	0.61	0.18		B
	N	7	2.13	1.12	A	
	N+P	7	1.56	1.55	A	B
	P	7	0.87	0.72		B

The control and N+P treatments were compared for all of the sites sampled during this project (Tables 7 and 8, respectively) using both the SNK and the WD tests. The SNK test is much more conservative with respect to Type 1 Errors than the WD test. The WD is used often in plot studies with high variability and low replicate numbers (n) [20]. The WD test distinguished 5 significant groups within the data in contrast to the 3 groups the SNK test distinguished. Site NB-02 was consistently higher than the other sites, suggesting significant periphytic biomass enrichment from the point source at Stephenville. The control treatment concentrations of 1.04 μg cm^-2 ^(s.d. = 0.74, n = 157) represents a reasonable estimator of baseline chlorophyll *a *productivity for the Bosque River watershed. The chlorophyll *a *values for the N+P treatments (MPP) were compared between all of the sites sampled (Table 8). The sites with the consistently highest MPP values were NB-02, NB-04, NB-05, and MB. There is clear evidence of differences across sites in periphytic response to nutrients over time (Tables 7 and 8). These data represent direct measures of the spatial and temporal variability of trophic status.

**Table 7:** Student-Newman-Keuls' (SNK) Test and Waller-Duncan K-Ratio (WD) T Test Comparison of Chlorophyll *a *concentrations for *Control treatments *using Passive Diffusion Periphytometers in the Bosque River Watershed for all sites sampled during the indicated periods. SNK and WD Groups represent values from discrete populations, when compared across all dates.

Date	Site	Number of Replicates	Mean Chl. *a *(ug cm^-2^)	Std Dev. (ug cm^-2^)	SNK Group (α = 0.05)			WD Group (α = 0.05)					
July 17 – 30, 1997	**NB-01**	10	0.88	0.60			C					E	F
	**NB-02**	10	4.58	0.86		B			B				
	**NB-04**	10	1.73	0.37			C			C	D	E	F
	**NC**	10	0.48	0.14			C			C			F
	**MB**	10	0.47	0.20			C			C	D		F
April 5 – 19, 1998	**NC**	10	0.71	0.26			C					E	F
	**MB**	10	0.56	0.27			C					E	F
May 11 – 26, 1998	**NB-01**	8	0.40	0.16			C					E	F
	**NB-02**	9	9.02	6.95	A			A					
	**NB-04**	9	2.50	0.70			C				D	E	
	**NB-05**	9	2.69	0.80			C					E	
	**HC**	7	1.46	0.48			C			C	D	E	F
	**MB**	8	0.83	0.13			C					E	F
July 30 – August 13, 1998	**NB-04**	8	0.49	0.24			C					E	F
	**NB-05**	9	0.99	0.26			C					E	F
October 10 – 24, 1998	**NB-01**	7	0.80	0.34			C					E	F
	**NB-02**	6	1.94	1.19			C			C	D	E	
	**NB-03**	7	0.61	0.18			C					E	F

**Table 8:** Student-Newman-Keuls' (SNK) Test and Waller-Duncan K-Ratio (WD) T Test Comparison of Chlorophyll *a *concentrations for *Nitrogen plus Phosphorous (N+P) treatments *using Passive Diffusion Periphytometers in the Bosque River Watershed for all sites sampled during the indicated periods. SNK and WD Groups represent values from discrete populations, when compared across all dates.

Date	Site	Number of Replicates	Mean Chl. *a *(ug cm^-2^)	Std Dev. (ug cm^-2^)	SNK Group (α = 0.05)					WD Group (α = 0.05)					
July 17 – 30, 1997	**NB-01**	10	0.98	0.53					E					E	F
	**NB-02**	10	5.19	1.86	A	B	C				B	C			
	**NB-04**	8	5.73	1.36	A	B					B	C			
	**NC**	10	2.67	0.82			C	D	E				D	E	
	**MB**	10	5.87	1.93	A	B					B	C			
April 5 – 19, 1998	**NC**	10	1.21	0.60					E					E	F
	**MB**	10	0.91	0.63					E					E	F
May 11 – 26, 1998	**NB-01**	9	0.37	0.13					E						F
	**NB-02**	8	8.07	6.69	A					A					
	**NB-04**	7	4.41	1.77		B	C	D				C	D		
	**NB-05**	8	6.13	2.54	A	B					B	C			
	**HC**	8	1.16	0.58					E					E	F
	**MB**	8	6.35	2.94	A	B				A	B				
July 30 – August 13, 1998	**NB-04**	9	4.55	1.92		B	C				B	C			
	**NB-05**	8	2.15	0.50				D	E					E	F
October 10 – 24, 1998	**NB-01**	6	0.73	0.20					E						F
	**NB-02**	6	1.50	1.09					E					E	F
	**NB-03**	7	1.56	1.55					E					E	F

### Nutrient gradient responses

A phosphorus enrichment gradient was deployed at sites NB-03 and NB-05 (sites previously deemed to be P limited, Table 5) to determine the critical concentration of biologically available phosphorus in the stream during the sampling period of July 30 – August 13, 1998. Site NB-05 was phosphorous limited (Table 5). A significant growth response was detected at the phosphorous concentration of P5 (500 μg/l PO^4^-P), but not at P4 (200 μg/l PO^4^-P). The other phosphorous concentrations did not elicit a growth response significantly different from the control. These results should not be interpreted the same as a dose-response nutrient enrichment assay, because it is reasonable to assume the algae on the periphytometer filter surface have greater access to the P molecules diffusing through the filter than those algal cells attached to rocks have to P in the water column. Thus the actual effective concentration of P that elicited the increased growth rate was not known.

Site NB-03 was co-limited; none of the phosphorous treatments elicited a discernible response greater than the control at this site. However, the N+P treatment resulted in a significant increase in biomass productivity (Tables 7 and 8). This suggests that while phosphorus was present in excess at the site (no increased productivity resulting from P treatments), the ratio of N to P was close to the threshold for limiting both nutrients. Adding just N or P did not elicit a significant increase in biomass over the two weeks, but N+P elicited a 10-fold increase in biomass productivity.

## Conclusion

The rivers and streams in the Lake Waco/Bosque River Watershed exhibited varying degrees of nutrient enrichment over the 18-month duration of this project. The North Bosque River at NB-02 consistently exhibited the highest degree of water quality impact due to nutrient enrichment. Sites NB-04 and NB-05 were the next most enriched sites. Chlorophyll *a *productivity on passive diffusion periphytometers in response to nutrient enrichment at multiple sites in late summer (July and August) versus spring (April and May) were significantly different (α = 0.05); thus we failed to reject the alternate hypotheses H_A1_: "Stream trophic status varied throughout the seasons across sites." The significance of this finding is that the Bosque River has been managed for phosphorus alone, while these conclusions indicate phosphorus is not the only nutrient affecting algal growth rates. Establishing a single nutrient criteria for N or P may not be adequate to protect water quality; rather, a season-specific and reach-specific nutrient criteria may be necessary.

Seasonality plays a significant role in the magnitude of the biological response to nutrients, suggesting seasonal differences in phosphorus assimilative capacity in the system. The highest assimilative capacity is in the late spring to early summer, while the lowest assimilative capacity was in the late fall. These results are similar to those of Stanley *et al.*[26] in Texas, and Matlock *et al. *[27] in Oklahoma. The LETSI is a reasonable method for assessing the response of periphyton in situ in complex watersheds.

The nutrient gradient treatments applied at two different sites demonstrated that algae responded to phosphorus enrichment progressively, thus we failed to reject **H**_**A2**_: "Periphytic chlorophyll *a *growth in response to nutrient enrichment from the passive diffusion periphytometer was independent of nutrient enrichment concentration." Passive diffusion periphytometers induce a nutrient growth response dependent upon the concentration of nutrient enriched media, and thus are a viable nutrient bio-assay for water quality management. These results are analogous to the results of algal growth potential studies in lakes and reservoirs [28].

These findings confirm previous findings that phosphorus biotic dynamics in streams are controlled by complex ecological processes that require spatial and temporal resolution to quantify and understand. In addition, these results support the emerging understanding that point source loadings of phosphorus are particularly problematic in streams due to their persistent nature – phosphorus is loaded to the stream at a constant rate, resulting in potential sorption to sediment in the stream banks and bed, and potential modification of nutrient spiraling throughout the system.

Management of stream water quality with regards to nutrient loads requires intensive characterization of seasonal sensitivity to nutrient loads to prevent algal biomass blooms and other deleterious effects from nutrient enrichment. Just measuring the trophic class of a water body (equivalent to the control response on the periphytometers) does not indicate the potential or sensitivity of the water body to nutrient enrichment. Passive diffusion periphytometers represent a potentially valuable method for estimating stream algae sensitivity to nutrient enrichment. However, for this approach to be quantitative we must have a better understanding of the relationship between the other variables that control algal growth: light, temperature, turbidity, grazing, and others.

## Abbreviations

LETSI Lotic ecosystem trophic status index

MPP Maximum potential productivity

N Nitrogen

NP Nitrogen plus phosphorus

P Phosphorus

SNK Student-Newman-Keuls' Test

WD Waller – Duncan K Ratio Test

WWTP Wastewater Treatment Plant

## Competing interests

The author(s) declare that they have no competing interests.

## Authors' contributions

AR collected primary data, conducted laboratory analyses, provided statistical analyses of the data, and drafted the manuscript as part of her thesis. MM designed the experiments, identified sites, provided technical and analytical support for field and laboratory work, participated in primary data collection and analysis, interpreted the data with AL, and revised the MS thesis into manuscript format. Both authors read and approved the final manuscript.
